# Servant Girls

**Published:** 1857-07

**Authors:** 


### SERVANT GIRLS.

A FAIR correspondent from one of the most beautiful towns of interior Pennsylvania, writes:

"I know of no place where ladies work harder and are so much enslaved by the hirelings of the kitchen, as in this quiet town of three thousand inhabitants. Six weeks ago I hired a girl, who recommended herself as being very capable of doing work and exceedingly fond of children. I soon found that her capabilities consisted mainly in washing, starching, and ironing her own dresses, of which she never had less than three, with half a dozen collars, in the weekly wash. The second day after she came to me, she walked into my room to use my hair-brush. I looked at her with astonishment. She next went to the wash-stand. 'Mary, there is a basin, towel, soap, and glass in your own room; these articles I do not use in common with any one.' 'Yes, but I like this soap of yours so much,' replied the 'capable' Mary.

"The first sabbath I allowed her to attend church, leaving at noon, to return before supper-time. At ten o'clock she had not come home, so I locked up the house and retired. An hour and a half later there were voices at the door, asking admittance, but they were not heeded. Next morning, about seven, my 'capable' Mary came in, with many reasons for not returning at the specified time. She always came home before night, after that. But she would not get up early enough in the morning. It was necessary for me to call her, and even then, seven o'clock usually found her in bed. This was intolerable, and I dismissed her. She is now in a minister's family, whose wife says, that 'Mary is poor help; she does very little; we must call her two or three times every morning—but what can we do, we are so dependent on our servant girls?'"

From this it would seem that there is rather more trouble in being suited as to help, in the rural districts, than in the city; but whether in city or country, it is not to be denied, that much of the worthlessness of our servants depends on the incompetency of their mistresses. The chief defects are a want of patience, firmness, and due consideration of their ignorance and infirmities. The best servants are those who are sternly held up to the fullest performance of their duty. But, while we do this, we should be considerate in our requirements, courteous in our bearing towards them, and prompt in our payments.

